# Acute Behavior of Oxygen Consumption, Lactate Concentrations, and Energy Expenditure During Resistance Training: Comparisons Among Three Intensities

**DOI:** 10.3389/fspor.2021.797604

**Published:** 2021-12-15

**Authors:** Gustavo A. João, Gustavo P. L. Almeida, Lucas D. Tavares, Carlos Augusto Kalva-Filho, Nelson Carvas Junior, Francisco L. Pontes, Julien S. Baker, Danilo S. Bocalini, Aylton J. Figueira

**Affiliations:** ^1^Department of Exercise Physiology Laboratory, Metropolitanas Unidas College, São Paulo, Brazil; ^2^Department of Translational Physiology Laboratory, São Judas Tadeu University, São Paulo, Brazil; ^3^Laboratory of Neuromuscular Adaptations to Strength Training, University of São Paulo, São Paulo, Brazil; ^4^Laboratory of Applied Sports Science, Institute of Physical Education and Sports, Federal University of Alagoas, Maceió, Alagoas, Brazil; ^5^Department of Evidence-Based Health, Brazilian Cochrane Center, University Federal de São Paulo, São Paulo, Brazil; ^6^Physical Activity and Aging Laboratory, School of Arts, Sciences and Humanities, University of São Paulo, São Paulo, Brazil; ^7^Centre for Health and Exercise Science Research, Hong Kong Baptist University, Kowloon Tong, Hong Kong, China; ^8^Experimental Physiology and Biochemistry Laboratory, Physical Education and Sport Center of Federal University of Espírito Santo, Vitoria, Brazil

**Keywords:** EPOC, energy expenditure (EE), caloric cost, resistance training (RT), strength training

## Abstract

**Purpose:** This study aimed to compare the oxygen consumption, lactate concentrations, and energy expenditure using three different intensities during the resistance training sessions.

**Methods:** A total of 15 men (22.9 ± 2.61 years) experienced in resistance training underwent 3 sessions composed of 8 exercises (chest press, pec deck, squat, lat pull-down, biceps curl, triceps extension, hamstring curl, and crunch machine), which were applied in the same order. The weight lifted differed among the sessions [high session: 6 sets of 5 repetitions at 90% of 1-repetition maximum (1-RM); intermediary session: 3 sets of 10 repetitions at 75% of 1-RM; and low session: 2 sets of 15 repetitions at 60% of 1-RM]. The oxygen consumption (VO_2_)—during and after (excess post-exercise oxygen consumption (EPOC)) the session, blood lactate concentration, and energy expenditure (i.e., the sum of aerobic and anaerobic contributions, respectively) were assessed.

**Results:** The VO2 significantly decreased in the function of the weight lifting (*F*_(2.28)_ = 17.02; *p* < 0.01; ${\text{η}}_{\text{G}}{}^{2}$ = 0.32). However, the aerobic contributions significantly increase in the function of the weight lifting (*F*_(2.28)_ = 79.18; *p* < 0.01; ${\text{η}}_{\text{G}}{}^{2}$ = 0.75). The anaerobic contributions were not different among the sessions (*p* > 0.05; ${\text{η}}_{\text{G}}{}^{2}$ < 0.01). Thus, the total energy expenditure during the session (kcal) significantly increased in the function of the weight lifting (*F*_(2.28)_ = 86.68; *p* < 0.01; ${\text{η}}_{\text{G}}{}^{2}$ = 0.75). The energy expenditure expressed in time unit (kcal·min^−1^) was higher in low session than in high session (*F*_(2.28)_ = 6.20; *p* < 0.01; ${\text{η}}_{\text{G}}{}^{2}$ = 0.15).

**Conclusion:** The weight lifted during resistance training-induced different physiological responses, which induced higher energy expenditure per unit of time during the low session.

## Introduction

Resistance training (RT) has been practiced by millions of people such as athletes and individuals interested in fitness, who seek increased health statuses, such as improving body composition, lipid profiles, and athletic performance *via* improvements in strength and muscle power (Nunez et al., 2019; Gonzalez-Hernandez et al., 2020).

Data on the metabolic profiles of resistance training responses of the practitioner to oxygen consumption (VO2), aerobic and anaerobic contributions, blood lactate concentration, and energy expenditure (EE) remain unclear in the literature.

However, RT induces several adaptations and requires different metabolic demands (aerobic and anaerobic) during the session (Schoenfeld, 2013; Aguiar et al., 2018). However, understanding better how the variables such as the number of sets and repetitions and the intensities (weight lifted) could contribute to the metabolic system that can help to prescribe resistance training methodologies with accuracy.

In this context, demonstrating how different RT prescriptions influence metabolic stress (i.e., energy contributions) is important in practical and study approaches. Identification of RT sessions with higher EE—during and after exercise is useful for health purposes and body weight management (Da Silva et al., 2010; Kelleher et al., 2010; Nunez et al., 2019). Considering the size principle of motor unit recruitment, the lower threshold fibers (type I) are activated during submaximal contractions, while the higher threshold fibers (type II) are progressively recruited dependent on function and intensity (Garber et al., 2011; Morton et al., 2019). Thus, an increase in the EE is expected with increased increments in the weight lifted. Little study has investigated resistance training and metabolic systems and their energy cost. Thornton and Potteiger (2002) reported that RT sessions using 2 sets of 8 repetitions at 85% of maximal repetition [1-repetition maximum (1-RM)] produce greater EE—mainly after exercise—over 2 sets of 15 repetitions at 45% of 1-RM.

Recently, Brunelli et al. (2019) investigated EE, energy system contributions after an acute low-load or high-load RT using two different loads: low-load RT (30% of 1-RM) or a high-load RT (80% of 1-RM) during knee extension exercise. Exercise EE and the anaerobic lactic system contributions were significantly higher for 30% of 1-RM compared to 80% of 1-RM.

Considering these results and the gap in the current literature, previous studies have not investigated intermediate intensities (for example, 75% of 1-RM), and more exercises per session, as are often performed in practical RT sessions. Our hypothesis is that an RT session consisting of 8 exercises, associated with intensities >80% of 1-RM, could promote an increase in EE.

Thus, the aim of this study was to compare EE using oxygen consumption values during and after RT sessions using intermediate (IS), high (HS), and low (LS) intensities, performed using 30 repetitions.

## Materials and Methods

### Participants

A total of 15 healthy young men with at least 12 months of experience in RT were recruited and assigned to a randomized trial (Table 1). All the procedures were approved by the Ethical Institutional Review Board of São Judas Tadeu University (protocol: 2.022.898) and conducted according to the principles expressed in the Declaration of Helsinki. The volunteers were told to refrain from any resistance exercise during the period of the experiment; all the participants read and signed an informed consent document (João et al., 2020).

**Table 1:** Participants baseline characteristics and dietary intake (mean ± SD).

**Age (years)**	**22.87, 2.61**
**Time experience (years)**	**3.27, 1.53**
**Body mass (kg)**	**83.60, 9.76**
**Height (cm)**	**183.07, 5.60**
**% Body Fat**	**9.94, 3.32**
**Fat mass (kg)**	**10.63, 2.62**
**Fat-free mass (kg)**	**72.97, 7.47**
**BMI (kg/cm^2^)**	**24.90, 2.15**
**RMR (kcal)**	**2395.47, 415.80**
**Carbohydrate (g)**	**482.00, 46.63**
**Protein (g)**	**156.87, 11.16**
**Lipids (g)**	**67.60, 7.41**
**Total (kcal)**	**3325.73, 245.15**

The following criteria were established as exclusion: (1) smokers; (2) submitted to diets designed for both the reduction of body mass and/or muscle mass increase; (3) free of any metabolic disorders with no medication intake that affects EE (sympathomimetics, bronchodilators, antidepressants, amphetamines, and illicit drugs); (4) muscle or tendon injury in the last 3 months; (5) being under the treatment of infectious disease; (6) having used in the last 6 months or be using any type of ergogenic agent of hormonal origin to increase strength or hypertrophy; and (7) no change in diet as reported by the food recall questionnaire. Inclusion criteria were as follows: (1) minimum experience of 12 months in RT; (2) reports of minimum training frequencies of 3 times per week; (3) present resting systolic blood pressure between 120 and 129 mm Hg and diastolic blood pressure between 80 and 84 mm Hg (Pescatello et al., 2004), assessed immediately before the experimental sessions through an auscultatory method; and (4) demonstration of a medical declaration that guarantees healthy clinical status to participate. All the individuals participating in this study answered the sociodemographic questionnaire with open questions about physical activity history, nutritional information, disease history, and family aspects.

### Experimental Design

**Figure 1:**
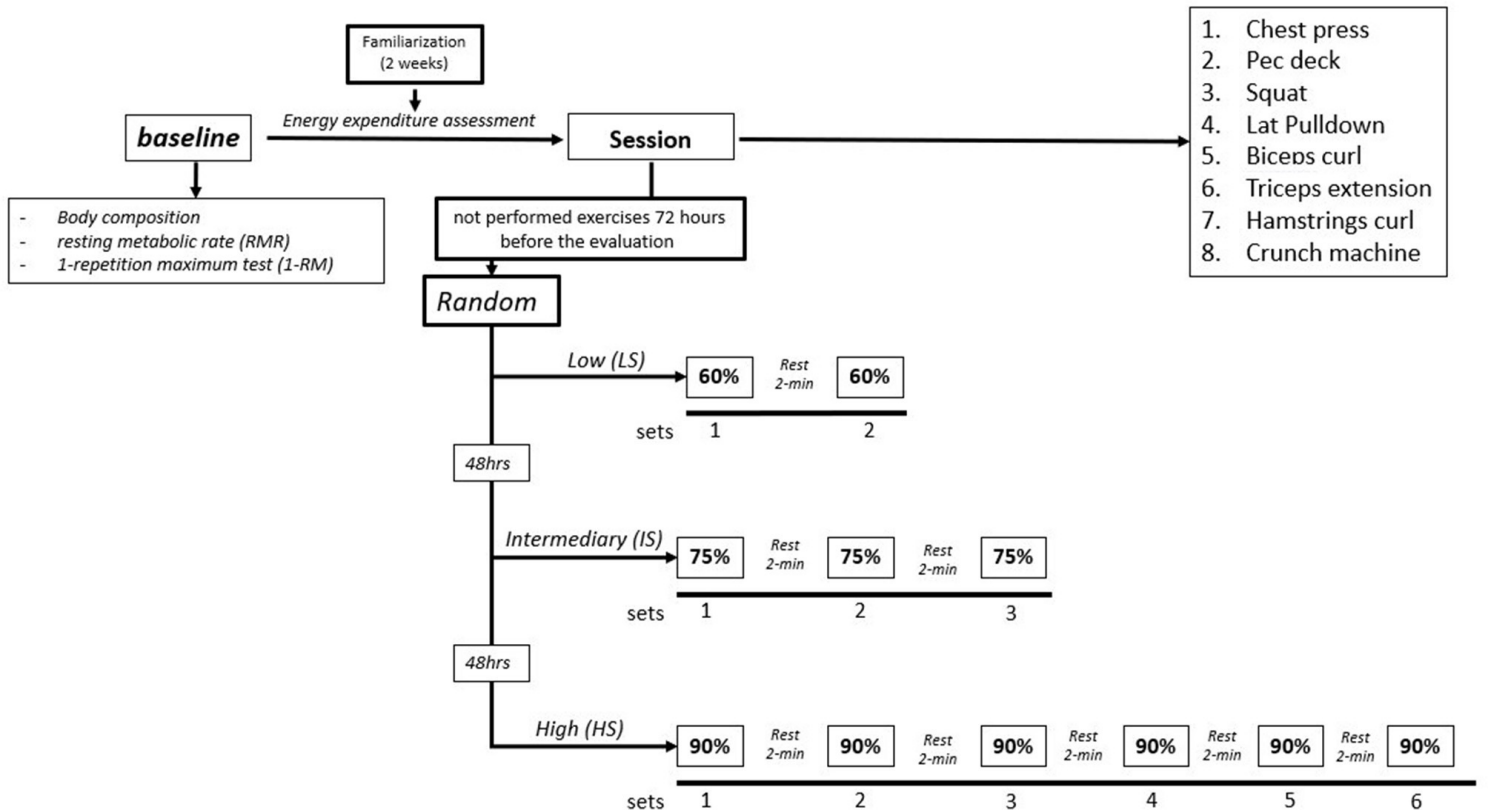
Experimental design.

The experimental design was composed of 6 visits. In the first visit, body composition and 1 RM were assessed. A familiarization with each exercise using the equipment and measurements (e.g., the mask of the gas analyzer) was performed during the second visit. In the third visit, the resting metabolic rate (RMR) was assessed for 30-min in a supine position. After 2 weeks, during the 3 lasted visits, participants underwent the experimental RT sessions composed of 8 exercises, which were performed at low (60% of 1-RM; LS), intermediary (75% of 1-RM; IS), and high (90% of 1-RM; HS) intensities. The choice of intensities used reflects most RT methods and was based on the American College of Sports Medicine (ACSM) and the National Strength and Conditioning Association (NSCA) recommendations (Fry, 2004; Fragala et al., 2019). The sessions were applied in random order and separated by 48 h of recovery (Figure 1).

In the familiarization session, the weight lifted was adjusted to perform 1 set of 20 repetitions. All the participants were informed about how to correctly perform the exercises and were under the direct supervision of the study team. The VO_2_ was monitored constantly and the blood lactate concentrations were obtained at rest and after experimental sessions (i.e., immediately and after 5, 10, 15, and 20 min of recovery).

The RT was strictly controlled so that individuals only exercised under the direct supervision of an experienced strength and conditioning professional. Individuals were not allowed to perform any other RT exercises during this period. All the participants were instructed not to consume coffee 12 h before the evaluations. In addition, all the participants did not exercise for at least 48 h prior to evaluation according to a study by Brigatto et al. (2019). All the subjects were monitored continuously using telephone calls and personal interviews to ensure adherence. No participants reported signals of muscle microtrauma (e.g., muscle pain) or conducting strenuous sessions 72 h before the experiments. Nutrient intake was assessed using a 24-h food recall on 2 non-consecutive weekdays and 1 weekend day.

### Body Composition

Body mass (BM) was measured using the G-Tech® (Hospital Medical Products – ACCUMED / serial: 47110100965, China) scale with an accuracy of 0.100 g with the individuals positioned barefoot and with minimum possible clothing. Height (H) was measured using a Sanny® stadiometer with a precision of 0.1 cm. BM index (BMI) was obtained using the same equation: BM/(H^2^) and circumferences were measured using a standard protocol outlined previously (Cavedon et al., 2018). To assess body composition and subcutaneous fat thickness, an ultrasound imaging unit was used (BodyMetrix® PRO System, Intelametrix, Livermore, California, USA—BodyView^TM^ software) with a wave frequency of 2.5 MHz (Selkow et al., 2011). The ultrasound probe was applied perpendicularly to the skin for measurement. A water-soluble gel was used on the transducer to help acoustic coupling and avoid excessive skin pressure. The individuals were instructed to fast for 3 h before testing. The pretest and posttest evaluations were performed at the same time. Imaging was performed on the right side of the bodies of individuals and to further ensure the accuracy of the assessments, at least 3 pictures were taken. The average of the 3 assessments was used for statistical analysis.

### 1-Repetition Maximum Test

Maximum dynamic strength was assessed using a 1-RM test for the following exercises: chest press, pec deck, squat, lat pull-down, biceps curl, triceps extension, hamstring curl, and crunch machine (fitness line equipment, GervaSport®, Spain), all accomplished in the pieces of equipment. The testing protocol followed previous recommendations by Haff and Triplett (Haff and Triplett, 2015). Participants reported to the laboratory having refrained from any exercise other than activities of daily living for at least 72 h before testing.

In brief, participants warmed up for 5 min on a treadmill (Movement Technology, São Paulo, Brazil) at 60% of maximum heart rate. During the first set, participants performed 5 repetitions at ~50% of the estimated 1-RM followed by 1 set of 3 repetitions at ~60–80% of the estimated 1-RM with a 3-min recovery interval between sets. After the warmup sets, participants had 5 attempts to find their 1-RM with 3-min intervals between trials. The 1-RM was deemed as the maximum weight that could be lifted no more than once using the correct technique. Verbal encouragement was given throughout the testing. All the testing sessions were supervised by the study team and deemed valid (Table 2).

**Table 2:** Test of 1 maximum repetition and load used in each exercise (mean ± SD).

	**Test**	**LS**	**IS**	**HS**
**Exercises**	**1-RM (kg)**	**60% of 1-RM (kg)**	**75% of 1-RM (kg)**	**90% of 1-RM (kg)**
Chest press	93 ± 23	56 ± 14	70 ± 17	84 ± 21
Pec deck	80 ± 19	48 ± 11	60 ± 14	72 ± 17
Squat	103 ± 27	62 ± 16	78 ± 20	93 ± 24
Lat pull-down	105 ± 19	63 ± 11	79 ± 14	94 ± 17
Biceps curl	47 ± 9	28 ± 60	35 ± 70	42 ± 90
Triceps extension	93 ± 13	56 ± 80	70 ± 10	84 ± 12
Hamstrings curl	111 ± 18	66 ± 11	83 ± 14	100 ± 17
Crunch machine	108 ± 14	65 ± 90	81 ± 11	97 ± 13

*Low (60% of 1-RM; LS), intermediary (75% of 1-RM; IS), and high-intensities (90% of 1-RM; HS)*.

The test-retest interclass correlation coefficient (ICC) and standard error of measurement (SEm) from our laboratory for 1-RM were applied to all the exercises. Therefore, test-retest ICC and SEm for 1-RM_chestpress_ were 0.996 [IC-95%: (0.986; 0.999)] with SEm (0.125) and test-retest for 1-RM_squat_ was 0.998 [IC-95%: (0.986; 0.999)] with SEm (0.031) (Table 2).

### Experimental Sessions

The participants performed a warm-up for 5 min before all the protocols on a treadmill (Movement Technology, São Paulo, Brazil) at 60% of maximum heart rate. After that, the participants performed 20 repetitions at 50% of 1-RM for each one of the exercises. After the warmup sets, the participants rested for 5 min and then started the protocols. All the sessions were composed of 8 exercises (chest press, pec deck, squat, lat pull-down, biceps curl, triceps extension, hamstrings curl, and crunch machine) performed sequentially. The participants in the sessions LS (60% of 1-RM) were encouraged to reach 15 repetitions in 2 sets, IS (75% of 1-RM) were encouraged to reach 10 repetitions in the 3 sets; and HS (90% of 1-RM) were encouraged to reach 5 repetitions in the 6 sets. The interest and interexercise rest was 120 s during all the protocols. The load was defined based on previous tests of a 1-RM. Thus, the load was not changed throughout the session.

In all the protocols, the cadence of repetitions was carried out in a controlled fashion, with phase concentric and eccentric muscle actions of ~1.5 s, for a total repetition duration of ~3 s (controlled by a metronome).

### Internal Responses

The VO_2_ during the RT sessions was measured using a gas analyzer (Fitmate Pro; COSMED®, Rome, Italy) with a flexible flowline as described previously (Lee et al., 2011). The gas analyzer was calibrated following the specifications of the manufacturer before each test. Immediately after completion of the exercise protocol, EPOC was measured for a total of 30 min in a supine position. The VO_2_ of the participants was obtained every 2 min. The area under the curve was assumed as the aerobic energy (AE) equivalent during the different experimental sessions. The integral was obtained through the software Origin (OriginPro 8.0, OriginLab Corporation, Microcal, Massachusetts, USA).

The lactate concentration was determined using a lactimeter model Accusport Plus Roche®, following the recommendation from previous studies (Foxdal et al., 1990; Franchini et al., 2004). Blood samples were taken from a finger capillary before the protocol (i.e., at rest); monitored immediately; and 5, 10, 15, and 20 min after RT session. To obtain anaerobic energy (ANE) equivalent, data were analyzed as the difference between peak and baseline values [LaΔ], using the equivalent of 3 ml·kg^−1^ of oxygen for each unit of lactate accumulated (Hunter and Byrne, 2005; Bertuzzi et al., 2016). The AE, ANE, and EPOC data were converted into calorie units using the equivalent of 5.05 calories (kcal) per liter of oxygen consumed (Phillips and Ziuraitis, 2003). Thus, the total energy equivalents were assumed as the sum of AE, ANE, and EPOC.

### Load Control

The “absolute load” for each exercise performed was calculated using the total weight lifted during the training period (i.e., no. of sets × no. of repetitions × weight lifted per repetitions) (Scott and Reis, 2016).

### Statistical Analyses

The sample size required was estimated using G^*^Power 3.1 software (version 3.1.9.4, Heinrich-Heine-University, Germany) (Faul et al., 2007), using effect sizes from a previous study comparing EE of different intensities during set RT (Scott et al., 2010). A priori power analysis assuming an estimating error of α = 0.05 and power = 80% to an actual power was 0.86 suggested a sample size of 8 participants to achieve a statistically significant difference between intensities. Thus, the use of *n* = 15 enabled a statistical power = 1.80.

However, 15 individuals were initially assigned to perform RT sessions with different intensities. Repeated measures ANOVA were used to analyze EE values, followed by Bonferroni's *post-hoc* test, where necessary. Normality, homogeneity, and sphericity assumptions were confirmed with Shapiro–Wilk, Levene's, and Mauchly‘s tests, respectively. Case of spherical (homogeneous) nonprincipal values used Greenhouse–Geisser correction. The generalized *eta* square (${\text{η}}_{\text{G}}{}^{2}$) was used as effect size and interpreted according to Cohen. For the CI for intraclass correlation, calculations using a (psychometric) package in the statistic program R software. Therefore, CI was then calculated at the desired level using formulas provided by McGraw and Wong (1996) and Bliese (2000). Overall data are presented as means, SDs, and 95% CI. Alpha was set at 0.05. The data were processed in R version 1.0.44 for Macintosh software.

## Results

The test-retest ICC and SEm from our laboratory for 1-RM were applied to the main exercises. Therefore, test-retest ICC and SEm for 1-RM_chestpress_ were 0.996 [IC-95%: (0.986; 0.999)] with SEm (0.125) and test-retest for 1-RM_squat_ was 0.998 [IC-95%: (0.986; 0.999)] with SEm (0.031).

Table 3 demonstrates the external responses of the different intensities in the RT. The absolute load was lower in the LS intensity when compared to IS and HS (*F*_(2.28)_ = 16.77; *p* < 0.01; ${\text{η}}_{\text{G}}{}^{2}$ = 0.40). However, there were no significant differences between IS and HS. Due to absolute load, there were observed significant differences among duration of the sessions (*F*_(2.28)_ = 2,773.34; *p* < 0.01; ${\text{η}}_{\text{G}}{}^{2}$ = 0.98).

**Table 3:** External responses during different experimental sessions prescribed at low (60% of 1-RM; LS), intermediary (75% of 1-RM; IS), and high-intensities (90% of 1-RM; HS) (mean ± SD).

**Parameters**	**LS**	**IS**	**HS**
Absolute-load (kg)	9858 ± 4526 [7352; 12365] ^*^^†^	15028 ± 2274 [13769; 16287]	17570 ± 4764 [14932; 20208]
Duration the session (min)	44.00 ± 3.02 [42; 46] ^*^^†^	61.40 ± 3.27 [59; 63] ^*^	116.00 ± 4.09 [113; 118]

*Mean ± SD, standard deviation; CI, confidence interval*.

*Absolute-load = no: of sets x no. of repetitions × weight lifted (kg)*.

* *p < 0.05 vs. HS (90% of 1-RM)*.

† *p < 0.05 vs. IS (75% of 1-RM)*.

Table 4 demonstrates the internal responses during the different intensities in RT. The VO_2_ was higher during LS when compared with other intensities (*F*_(2.28)_ = 17.02; *p* < 0.01; ${\text{η}}_{\text{G}}{}^{2}$ = 0.32). There were no significant differences in VO_2_ during the different intensities of HS when compared to IS. There were no significant differences in lactate concentrations between intensities of RT (*F*_(2.28)_ = 0.25; *p* = 0.77; ${\text{η}}_{\text{G}}{}^{2}$ = 0.01). There was a significant difference in AE in the HS when compared with LS and IS (*F*_(2.28)_ = 79.18; *p* < 0.01; ${\text{η}}_{\text{G}}{}^{2}$ = 0.75). There were no significant differences in the ANE (L) among intensities during sessions (*F*_(2.28)_ = 0.38; *p* = 0.68; ${\text{η}}_{\text{G}}{}^{2}$ = 0.01). The EPOC values were significantly lower in IS when compared LS and HS (*F*_(2.28)_ = 5.57; *p* < 0.01; ${\text{η}}_{\text{G}}{}^{2}$ = 0.12).

**Table 4:** The outcome of the lactate, energy expenditure and EPOC during different experimental sessions prescribed at low (60% of 1-RM; LS), intermediary (75% of 1-RM; IS) and high-intensities (90% of 1-RM; HS) (mean ± SD).

**Parameters**	**LS**	**IS**	**HS**
*Internal responses*			
VO_2_ (L^.^min^−1^)	1.61 ± 0.26 [1.47; 1.75] ^*^^†^	1.38 ± 0.32 [1.20; 1.56]	1.15 ± 0.25 [1.01; 1.29]
LaΔ **(**mmol^.^L^−1^**)**	8.85 ± 2.74 [7.33; 10.37]	8.03 ± 3.16 [6.28; 9.78]	8.69 ± 3.67 [6.66; 10.72]
*Energy equivalents*			
AE (L)	42.80 ± 6.70 [39.09; 46.51] ^*^^†^	59.25 ± 15.12 [50.88; 67.62] ^*^	107.12 ± 22.83 [94.48; 119.76]
ANE (L)	2.21 ± 1.01[1.65; 2.77]	1.99 ± 0.70 [1.61; 2.37]	2.20 ± 0.63 [2.17; 2.23]
EPOC (L)	11.14 ± 2.40 [9.82; 12.46] ^†^	10.01 ± 1.69 [9.07; 10.95] ^*^	11.52 ± 0.69 [11.14; 11.90]
Total (L)	56.16 ± 7.32 [52.11; 60.21] ^*^^†^	71.25 ± 15.48 [62.68; 79.82] ^*^	120.84 ± 22.83 [108.20; 133.48]

*Mean ± SD, standard deviation; CI, confidence interval; LaΔ, difference between lactate peak and baseline; AE, aerobic energy equivalent; ANE, anaerobic energy equivalent; EPOC, excess post-exercise oxygen consumption. Total = AE (L) + ANE (L) + EPOC (L)*.

* *p < 0.05 vs. HS (90% of 1-RM)*.

† *p < 0.05 vs. IS (75% of 1-RM)*.

**Figure 2:**
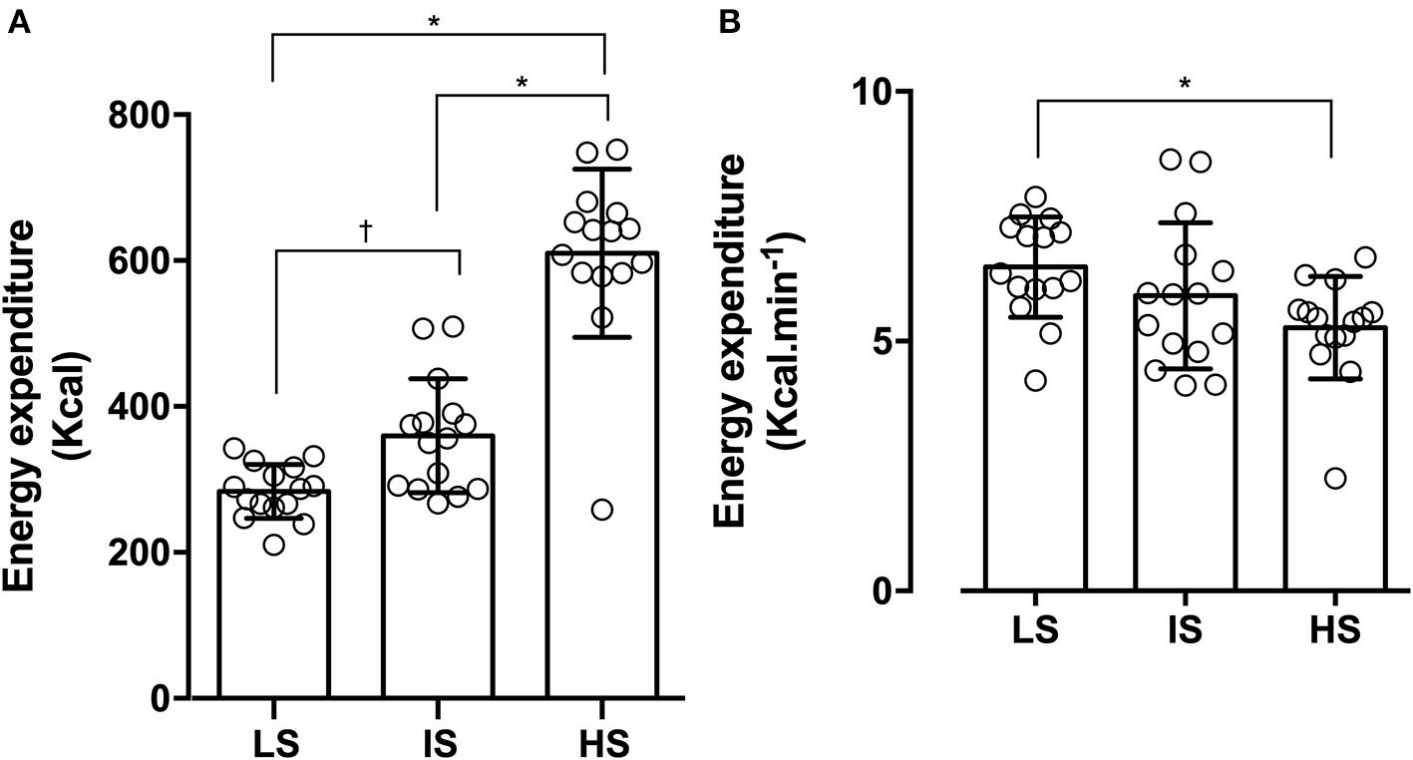
**(A)** Energy expenditure and **(B)** energy expenditure during different experimental sessions prescribed at low (60% of 1-RM; LS), intermediary (75% of 1-RM; IS) and high-intensities (90% of 1-RM; HS). *p< *0.05 vs*. HS (90% of 1-RM)^†^*p*< *0.05 vs*. IS (75% of 1-RM).

The EE of intensities is given in Figure 2. A significant difference in total EE (kcal) was found between all the intensities (*F*_(2.28)_ = 86.68; *p* < 0.01; ${\text{η}}_{\text{G}}{}^{2}$ = 0.75). Additionally, significant differences were found in EE expressed per time unit (kcal·min^−1^) only between LS (6.49 ± 1.01) and HS (5.27 ± 1.03) (*F*_(2.28)_ = 6.20; *p* < 0.01; ${\text{η}}_{\text{G}}{}^{2}$ = 0.16).

## Discussion

The purpose of this study was to compare the oxygen consumption, lactate concentrations, and EE using three different intensities (LS, IS, and HS) during the RT sessions. The main results demonstrated that HS presented higher values of EE, which was accompanied by a greater duration of the session and higher values of AE. However, when EE during sessions was expressed per time unit (kcal·min^−1^) of the session, higher values emerged during LS when compared with the HS. In addition, although the weight lifted had been different, the ANE was similar among the sessions.

According to the previous studies (Hunter et al., 2003; Haddock and Wilkin, 2006; Reis et al., 2017; Martorelli et al., 2021), the total EE increases when higher weights were lifted during the sessions. Hunter et al. (2003) compared EE using low (25% of 1-RM) or moderate intensities (65% of 1-RM) prescribed with the same total time (29 min) demonstrating that energy expenditure was 45% higher during the moderate-intensity session. Some of the studies use fixed repetitions during the sets, which limited direct comparisons of our results, mainly because the length of sessions acts as an inherent variable of the EE assessments.

Brunelli et al. (2019) demonstrated higher EE during the exercise performed at low-intensity (30% of 1-RM) compared with high-intensity (85% 1-RM) sessions. These results were accompanied by higher contributions from the anaerobic lactic pathway, indicating that the longest time in exercise compensated for the differences in the weight lifted during the sessions. Although the abovementioned article used only the knee extension machine, our results used 8 exercises and also demonstrated the higher values of EE per min during the LS compared with the HS. However, we could not demonstrate significant differences in ANE values, indicating that the higher values of EE in the LS were associated with the length of the session—that directly influenced the area of VO_2_ and not with changes in metabolic balance. A possible explanation of these differences between our results and those observed by Brunelli et al. (2019) may be the physical conditioning level of the participants. A study by Brunelli et al. (2019) was conducted with men with an inactive lifestyle who had not participated in regular resistance exercise programs in the past 12 months. On the other hand, in this study, one of the inclusion criteria of the participants was to have a minimum experience of 12 months in the RT. However, direct comparisons between these studies are limited because we did not use knee extension during our sessions and our RT sessions consisted of 8 exercises, while a study by Brunelli et al. (2019) only used 1 exercise modality.

The magnitude of the EPOC was measured for 30 min after the session, mainly considering that the participants returned to baseline approximately within 25 min (Farinatti et al., 2009; Ratamess et al., 2014). The EPOC may be the result of energy-requiring processes such as the replenishment of myoglobin oxygen stores, adenosine triphosphate, creatine phosphate, increased ventilation, heart rate, body temperature, triglyceride or fatty acid cycling, substrate utilization shifts from carbohydrates to fats, and mitochondrial activity (Borsheim and Bahr, 2003; Abboud et al., 2013). Our results indicated that the weight lifted between sessions can influence differently in these processes and corroborated previous studies using RT (Haddock and Wilkin, 2006; Farinatti et al., 2009; Abboud et al., 2013; Ratamess et al., 2014). On the other hand, in addition to the weight lifted, the session duration could cause the biggest physiological disorders induced by HS, related to the EPOC values. A study by Abboud et al. (2013) showed that during RT with a high absolute load (20,000 kg), the participants expended significantly more energy (484 ± 29 kcal) than the low absolute load (10,000 kg) (247 ± 18 kcal) and supported this study.

The methods to estimate the EEs may also contribute to value variation. In this context, this study used the area under the curve of the VO_2_ and lactate accumulation to estimate the AE and ANE, respectively.

Although these procedures were previously used in the context of RT, Brunelli et al. (2019) and Zagatto et al. (2016) demonstrated their validity and applicability and some limitations should be noticed in this study. First, it is preferable to use breath-by-breath assessments of VO_2_ to estimate the AE, allowing measurements with high precision during effort and recovery (here the VO_2_ was assessed through means every 2 min.). Second, this device limitation also precludes the determination of the anaerobic lactic equivalents through the fast phase of the EPOC, underestimating the total anaerobic equivalents. Third, although no differences were observed for ANE, the determination of lactate concentrations only after the session may underestimate the EEs, mainly because of lactate removal during recovery intervals. Therefore, collections after each exercise would be more accurate, as lactate would not be removed during recovery and, thus, the contribution would be greater. Four, an intense workout of 48 h before the evaluation could influence the performance of the participants because of muscle microtrauma. A low intensity workout of 48 h before will not influence the performance of the participants. We monitor the exercise routines 48 h before our experiments, as previously used for the same purpose (Brigatto et al., 2019). However, no participants reported signs of muscle microtrauma (e.g., muscle pain) or strenuous sessions 72 h before the experiments.

Together these methodological limitations possibly underestimated the AE, ANE, and EPOC values of the different RT sessions, it directly limits comparisons with previous studies. Even considering these limitations and the differences between studies, the results of this study seemed to be within the range of EE previously reported. In addition, this study used the absolute load method to determine the training load, which does not consider all the training variables (e.g., repetition velocity). We opted to use the absolute load method, mainly considering its practical application, outlined in a previous study (Scott and Reis, 2016). Future studies should advance the knowledge related to energy systems contribution using more robust approaches to equalize the absolute loads of the sessions.

## Conclusion

The results of this study advance the knowledge related to EE during the RT, demonstrating that when the EE during sessions was expressed per time unit (kcal·min^−1^) of the session, the LS presented higher values than HS. Thus, our results indicated that the duration of the exercise session—therefore the session length—induced greater influence on EE values than different energy balances (e.g., higher anaerobic contributions), at least when the sets were performed without fixed repetitions.

## Practical Application

In the context of applied practice, this information can be valuable for athletes, exercise physiologists, enthusiasts, and fitness professionals who know and understand with accuracy the contribution of the metabolic system during the RT sessions consisting of 8 exercises and different intensities (low, intermediate, and high). As a result, individuals can select the best option to achieve specific outcomes, for example, losing weight, gaining muscle mass, or a combination of both options.

## Data Availability Statement

The original contributions presented in the study are included in the article/supplementary material, further inquiries can be directed to the corresponding author/s.

## Ethics Statement

The studies involving human participants were reviewed and approved by Ethical Institutional Review Board of São Judas Tadeu University (protocol: 2.022.898). The patients/participants provided their written informed consent to participate in this study.

## Author Contributions

GJ and NC performed the work's statistics. JB, AF, DB and CK-F developed the manuscript. GA and FP conducted article researches and data collection. GA and GJ structured the study design. All authors of the research had an important contribution to carry out the study.

## Conflict of Interest

The authors declare that the research was conducted in the absence of any commercial or financial relationships that could be construed as a potential conflict of interest.

## Publisher's Note

All claims expressed in this article are solely those of the authors and do not necessarily represent those of their affiliated organizations, or those of the publisher, the editors and the reviewers. Any product that may be evaluated in this article, or claim that may be made by its manufacturer, is not guaranteed or endorsed by the publisher.

## Correction note

A correction has been made to this article. Details can be found at: 10.3389/fspor.2026.1813016.
