# Correction: Acute behavior of oxygen consumption, lactate concentrations, and energy expenditure during resistance training: comparisons among three intensities

**DOI:** 10.3389/fspor.2026.1813016

**Published:** 2026-03-13

**Authors:** Gustavo A. João, Gustavo P. L. Almeida, Lucas D. Tavares, Carlos Augusto Kalva-Filho, Nelson Carvas Junior, Francisco L. Pontes, Julien S. Baker, Danilo S. Bocalini, Aylton J. Figueira

**Affiliations:** 1 Department of Exercise Physiology Laboratory, Metropolitanas Unidas College, São Paulo, Brazil; 2 Department of Translational Physiology Laboratory, São Judas Tadeu University, São Paulo, Brazil; 3 Laboratory of Neuromuscular Adaptations to Strength Training, University of São Paulo, São Paulo, Brazil; 4 Laboratory of Applied Sports Science, Institute of Physical Education and Sports, Federal University of Alagoas, Maceió, Brazil; 5 Department of Evidence-Based Health, Brazilian Cochrane Center, University Federal de São Paulo, São Paulo, Brazil; 6 Physical Activity and Aging Laboratory, School of Arts, Sciences and Humanities, University of São Paulo, São Paulo, Brazil; 7 Centre for Health and Exercise Science Research, Hong Kong Baptist University, Kowloon Tong, Hong Kong, China; 8 Experimental Physiology and Biochemistry Laboratory, Physical Education and Sport Center of Federal University of Espírito Santo, Vitoria, Brazil

**Keywords:** EPOC, energy expenditure (EE), caloric cost, resistance training (RT), strength training

The reference “João GA, Rodriguez D, Tavares LD, Rica RL, Júnior NC, Reis VM, et al. Energy expenditure estimation of a moderate-intensity strength training session. Cogent Medicine (2020) 7(1). doi: 10.1080/2331205x.2020.1794500” was not cited in the article. The citation has now been inserted in the section **Materials and Methods**, *Participants*, Paragraph 1, and should read:

“A total of 15 healthy young men with at least 12 months of experience in RT were recruited and assigned to a randomized trial (Table 1). All the procedures were approved by the Ethical Institutional Review Board of São Judas Tadeu University (protocol: 2.022.898) and conducted according to the principles expressed in the Declaration of Helsinki. The volunteers were told to refrain from any resistance exercise during the period of the experiment; all the participants read and signed an informed consent document (João et al., 2020).”

The original version of this article has been updated.

## Publisher's note

All claims expressed in this article are solely those of the authors and do not necessarily represent those of their affiliated organizations, or those of the publisher, the editors and the reviewers. Any product that may be evaluated in this article, or claim that may be made by its manufacturer, is not guaranteed or endorsed by the publisher.

## Disclaimer

This is a transparency statement to clarify the use of the same dataset in publications by this research group published in *Cogent Medicine* (2020) and *Frontiers in Physiology* (2021).

Both articles originate from the doctoral thesis of Gustavo Allegretti João, which investigated energy expenditure during resistance training sessions in adults using indirect calorimetry and extrapolation methods. At the time of data collection, this approach was innovative and aimed at developing robust methodological tools to estimate caloric expenditure during full-session resistance training.

The study published in *Cogent Medicine* (2020) had as its primary objective the quantification of energy expenditure during a resistance training session composed of eight exercises performed at moderate intensity (60–75% of one-repetition maximum, 1RM), as well as the development and validation of a predictive regression equation to estimate caloric expenditure during complete resistance training sessions. The selection of this intensity range was based on its ecological validity and practical applicability, providing professionals with a novel and useful tool for real-world training contexts.

In contrast, the article published in *Frontiers in Physiology* (2021) addressed a distinct scientific objective, focusing on a comparative analysis of caloric expenditure across three different training intensities (60%, 75%, and 90% of 1RM), with training volume strictly equalized. This study aimed to examine the influence of training intensity, total training volume, and inter-set recovery intervals on energy expenditure, thereby extending the application of the previously validated equation to different training conditions.

Although both publications used the same sample derived from the doctoral thesis, the data were analyzed under different scientific questions, methodological designs, and analytical perspectives. Therefore, the identical results observed at 75% of 1RM in both articles are expected, as they originate from the same underlying dataset. This overlap does not constitute data duplication, but rather reflects the legitimate and widely accepted reuse of a dataset to address distinct research objectives.

The authors acknowledge that the earlier publication should have been explicitly cited in the *Frontiers in Physiology* article to enhance scientific transparency. The authors recognize this omission and appreciate the opportunity to clarify the relationship between the two studies.

In summary, the 2020 publication focused on the development and validation of a predictive model for estimating caloric expenditure, whereas the 2021 publication applied this model to compare different resistance training intensities. Despite sharing the same dataset, the studies are conceptually and methodologically distinct.

The authors remain at your disposal for any further clarification and trust that this statement adequately addresses the concerns raised.

